# Evolution of intermediate latency strategies in seasonal parasites

**DOI:** 10.1093/jeb/voae009

**Published:** 2024-02-08

**Authors:** Hannelore MacDonald, Dustin Brisson

**Affiliations:** Department of Biology, University of Pennsylvania, Philadelphia, PA, United States; Institute for Integrative Biology, ETH Zürich, Zürich, Switzerland; Department of Biology, University of Pennsylvania, Philadelphia, PA, United States

**Keywords:** evolution, seasonality, phenology, parasite

## Abstract

Traditional mechanistic trade-offs between transmission and parasite latency period length are foundational for nearly all theories on the evolution of parasite life-history strategies. Prior theoretical studies demonstrate that seasonal host activity can generate a trade-off for obligate-host killer parasites that selects for intermediate latency periods in the absence of a mechanistic trade-off between transmission and latency period lengths. Extensions of these studies predict that host seasonal patterns can lead to evolutionary bistability for obligate-host killer parasites in which two evolutionarily stable strategies, a shorter and longer latency period, are possible. Here we demonstrate that these conclusions from previously published studies hold for non-obligate host killer parasites. That is, seasonal host activity can select for intermediate parasite latency periods for non-obligate killer parasites in the absence of a trade-off between transmission and latency period length and can maintain multiple evolutionarily stable parasite life-history strategies. These results reinforce the hypothesis that host seasonal activity can act as a major selective force on parasite life-history evolution by extending the narrower prior theory to encompass a greater range of disease systems.

## Introduction

The timing of seasonal activity, or phenology, is an environmental condition affecting all aspects of life cycles including reproduction, migration, and diapause (Elzinga et al., 2007; Forrest & Miller-Rushing, 2010; Park, 2019; Pau et al., 2011; Lustenhouwer et al., 2018; Novy et al., 2013). The timing and prevalence of transmission opportunities for parasites, which could alter parasite life-history strategies, are also impacted by the phenology of host species (Altizer et al., 2006; Biere & Honders, 1996; Gethings et al., 2015; Hamer et al., 2012; MacDonald et al., 2020; Martinez, 2018; McDevitt-Galles et al., 2020; Ogden et al., 2018). For example, phenological patterns that extend the time period between when hosts are infected and when transmission occurs are expected to select for longer parasite latency periods (time between infection and new parasite release), as observed in some malaria species (*Plasmodium vivax*). In these systems, shorter latency period strains persist in regions where mosquitoes are present year-round, while longer latency period strains are more common in regions where mosquitoes are nearly absent during the dry season (White, 2011).

Recent theoretical work predicts that seasonal host activity can select for intermediate latency periods in monocyclic (one infectious cycle per season), obligate-killer parasites even when traditional mechanistic trade-offs between transmission and latency are omitted (MacDonald et al., 2022). In these systems, the optimal latency strategy is determined by host phenological patterns: Longer seasons select for longer periods between infection and the release of new parasites. While these results suggest that seasonal host activity patterns can serve as a selective driver of intermediate latency periods, they were only investigated in monocyclic, obligate-killer parasites. An extension of this work demonstrated that seasonal host activity can select for both a monocyclic parasite strategy (one round of infection per season, thus the same optimum from MacDonald et al. 2022) and a polycyclic parasite strategy (multiple rounds of infection per season) (MacDonald & Brisson, 2023) where host phenology dictates the optimal strategy. The theory developed thus far for the impact of host phenology on parasite evolution applies only to obligate-killer parasites, which, while numerous, only represent a small proportion of the vast diversity of parasite strategies in nature.

There is reason to expect that some of the main conclusions from prior studies on the impact of host phenology on parasite latency period evolution will apply to parasites that are not obligate killers. For example, all parasites must complete a latency period between infection and the release of parasite progeny, regardless of whether progeny release requires host death. Thus, selection on latency periods may operate similarly for all parasites in seasonal environments as releasing parasite progeny too early or too late is maladaptive if it mistimes interactions with seasonally available hosts. These studies suggest that host phenology could create important selective pressures affecting parasite latency period evolution in many seasonal disease systems.

Here we investigate the impact of seasonal host activity on latency period evolution for parasites not constrained to the obligate-killer lifestyle. We examine how two components of host phenology, the timing and duration of host emergence, impact parasite latency period evolution in non-obligate killer parasites. We demonstrate that the conclusions from previously published theory investigating the impact of host seasonal patterns on obligate-killer parasite evolution hold for non-obligate killer parasites. That is, seasonal host activity can select for both an intermediate latency period in the absence of a mechanistic trade-off between transmission and virulence and generate evolutionary bistability. These results demonstrate that host seasonal activity could serve as a major driver of parasite evolution in a wide range of parasite species.

## Materials and Methods

We modify a published model that studies how host phenology impacts the evolution of the time between infection and host death in an obligate-killer parasite (MacDonald et al., 2022) to study how host phenology impacts the evolution of non-obligate killer parasite latency periods (time between infection and the beginning of new parasite release, τ). The main modification between the previous model and this study is that the parasite does not kill its host to release progeny. Instead, hosts experience infection-induced virulence either as a reduction in fecundity or an increased mortality rate following infection. A second model in the present article also relaxes the assumption that new parasite release is synchronous after a set latency period (τ). Instead, infected hosts move to the infectious class (i) where they release new parasite progeny at a constant rate until they recover.

**Figure 1.**
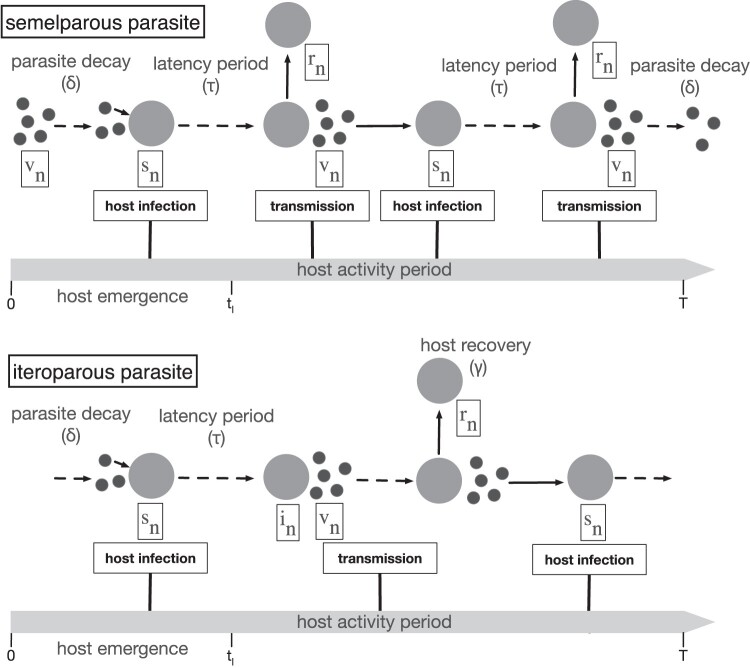
Diagrammatic representation of the infectious cycle within each season. All parasites (v) emerge at the beginning of the season (t=0), while all hosts (s) emerge at a constant rate between time t=0 and $t=t_{l}$. At time τ postinfection, parasite progeny (v) are released into the environment where they decay from exposure at rate δ. The top infection diagram shows the semelparous parasite model in which all parasite progeny released at time τ following infection at which point hosts recover to stage r. The bottom infection diagram shows the iteroparous parasite model in which parasites are released once infected hosts have entered the infectious stage (i) at time τ postinfection and continue to be transmitted until hosts recover to stage r at rate γ. If τ is less than half the season length, a second generation of infections can occur within the season. Parasite progeny that survive in the environment to the end of the season comprises the parasite population that emerge in the following season ($v\,\left(T\right)=\hat{v}\,\left(n+1\right)$).

The models describe the transmission dynamics of a free-living parasite that infects a seasonally available host (Figure 1). The exact disease system is left general, so it can be adapted to any system. Hosts, s, have non-overlapping generations and are alive for one season. The susceptible host cohort, $\hat{s}\,\left(n\right)$, enters the system at the beginning of the season. $\hat{s}\,\left(n\right)$ is a function of the number of uninfected hosts at t=T in season n−1. The parasite, v, must infect and release new infectious progeny before the end of the season to leave progeny in the environment to infect the next season’s host cohort. In the first model, parasites are semelparous; thus, infected hosts release all new parasite progeny synchronously. Parasite release occurs after a set latency period (τ) after which infected hosts move to the recovered class (r). In the second model, parasites are iteroparous; thus, new parasite progeny transmission is distributed over time. In this case, infected hosts move to the infectious class (i) after a set latency period at which point they release new parasite progeny at a constant rate until they recover to r. The number of rounds of infection the parasite completes within a season depends on τ. If there is a long period between infection and progeny release, the parasite completes one round of infection per season and is therefore monocyclic. If there is a short period between infection and progeny release, the parasite can complete multiple rounds of infection per season and is therefore polycyclic.

The duration of each season extends from t=0 to t=T. Time units are not specified in order to maintain the generality of the model across disease systems. It is expected that the relevant time unit will be in months for many disease systems, corresponding to spring and summer (Baltensweiler et al., 1977; Danks, 2006; Donovan, 1991; Grant & Shepard, 1984; Takasuka & Tanaka, 2013) and weeks for other disease systems (Cummins et al., 2011; Dalen, 2013; Danks, 2006). The initial conditions at the beginning of the season are $s_{n}\,\left(0\right)=0,v_{n}\,\left(0\right)=\hat{v}\,\left(n\right)=v_{n-1}\,\left(T\right)$ where $\hat{v}\,\left(n\right)$ is the size of the starting parasite population introduced at the beginning of season n determined by the number of parasite progeny remaining at the end of season (t=T) in season n−1. In the model with semelparous parasite release, the transmission dynamics in season n are given by the following system of delay differential equations (all parameters are described in Table 1):

**Table 1.** Model parameters and their respective values.

Parameter	Description	Value
$s_{n}$	Susceptible hosts	State variable
$v_{n}$	Parasites	State variable
$i_{n}$	Infectious hosts	State variable
$r_{n}$	Recovered hosts	State variable
$\hat{v}\,\left(n\right)$	Starting parasite population in season n	State variable
$\hat{s}\,\left(n\right)$	Host cohort in season n	State variable, $10^{7}$ when constant
$t_{l}$	Length of host emergence period	Time (varies)
T	Season length	Time (varies)
β	Transmission rate	$10^{-7}$ /time
α	Number of parasites released	200 Parasites when constant
δ	Parasite decay rate in the environment	1/time
$\mu_{s}$	Susceptible host death rate	0.25/time
$\mu_{i}$	Infectious host death rate	Varies, 0.25/time when constant
$\mu_{r}$	Recovered host death rate	Varies, 0.25/time when constant
γ	Infectious host recovery rate	3/time
τ	Latency period	Time (evolves)
ϕ	Reduction in host fecundity from infection	Varies, 1 or 0.5 when constant
σ	Host fecundity	200 hosts
ρ	Density-dependent parameter	0.0001
b	Trade-off parameter	75 for semelparous parasites, 200 for iteroparous parasites


$$\frac{d\,s_{n}}{d\,t}=\hat{s}\,\left(n\right)\,g\,\left(t,t_{l}\right)-\mu_{s}\,s_{n}\,\left(t\right)-\beta \,s_{n}\,\left(t\right)\,v_{n}\,\left(t\right),$$ (1a)

$$\frac{d\,r_{n}}{d\,t}=\beta \,e^{-\mu_{i}\,\tau }\,s_{n}\,\left(t-\tau \right)\,v_{n}\,\left(t-\tau \right)-\mu_{r}\,r_{n}\,\left(t\right),$$ (1b)

$$\frac{d\,v_{n}}{d\,t}=\beta \,\alpha \,\left(\tau ,b\right)\,e^{-\mu_{s}\,\tau }\,s_{n}\,\left(t-\tau \right)\,v_{n}\,\left(t-\tau \right)-\delta \,v_{n}\,\left(t\right)-\beta \,s_{n}\,\left(t\right)\,v_{n}\,\left(t\right).$$ (1c)

where $\mu_{s}$ is the susceptible host death rate, $\mu_{r}$ is the recovered host death rate, δ is the decay rate of parasites in the environment, β is the transmission rate and τ is the latency period. α is the total number of parasites released. In most cases, we assume α is a function of τ and the scaling parameter b, but also investigate the impact of a constant, trade-off free α.

When there is a trade-off between latency period (τ) and the number of parasite progeny released (α), we assume that the number of new progeny released increases as the latency period increases: $\alpha \,\left(\tau \right)=b\,{\left(\tau +0.5\right)}^{0.8}$. Note that when there is no trade-off between α and τ, the parasite growth rate in the host is essentially the trait under selection. That is, α is constant regardless of τ; thus, the trait that is effectively evolving is the rate that new parasites are assembled in between infection and host death (e.g., longer τ corresponds to slow assembly of new parasites).

In the model with iteroparous new parasite release, the transmission dynamics in season n are given by the following system of delay differential equations (again all parameters are described in Table 1):


$$\frac{d\,s_{n}}{d\,t}=\hat{s}\,\left(n\right)\,g\,\left(t,t_{l}\right)-\mu_{s}\,s\,\left(t\right)-\beta \,s_{n}\,\left(t\right)\,v_{n}\,\left(t\right),$$ (2a)

$$\frac{d\,i_{n}}{d\,t}=\beta \,e^{-\mu_{s}\,\tau }\,s_{n}\,\left(t-\tau \right)\,v_{n}\,\left(t-\tau \right)-i_{n}\,\left(t\right)\,\left(\mu_{i}+\gamma \right),$$ (2b)

$$\frac{d\,r_{n}}{d\,t}=\gamma \,i_{n}\,\left(t\right)-\mu_{r}\,r_{n}\,\left(t\right),$$ (2c)

$$\frac{d\,v_{n}}{d\,t}=\alpha \,\left(\tau ,b\right)\,i_{n}\,\left(t\right)-\delta \,v_{n}\,\left(t\right)-\beta \,s_{n}\,\left(t\right)\,v_{n}\,\left(t\right).$$ (2d)

where γ is the rate at which infected hosts recover from the infection.

The emergence phenology of hosts is captured by the function $g\,\left(t,t_{l}\right)$, which is a probability density that describes the per-capita host emergence rate through the timing and length of host emergence. We use a uniform distribution for simplicity, although other distributions are expected to have qualitatively similar results (MacDonald et al., 2020). Note that the uniform distribution used here translates to hosts emerging at a constant rate (equal to $1/t_{l}$)


$$g\,\left(t,t_{l}\right)=\left\{ \begin{array}{cc} \frac{1}{t_{l}} & 0\leq t\leq t_{l}\\ 0 & t_{l}<t\leq T \end{array}\right.$$

$t_{l}$ denotes the length of the host emergence period and T denotes the season length. The season begins ($t_{0}=0$) with the emergence of the susceptible host cohort ($\hat{s}\,\left(n\right)$) over the duration of $0\leq t\leq t_{l}.$

v⁢(T) parasites remaining in the system at the end of the season give rise to the next season’s initial parasite population ($\hat{v}\,\left(n\right)=v\,\left(0\right)$). Parasites only release progeny during the season (no further progeny release after t=T). Background mortality arises from predation or some other natural cause. We assume that infected hosts that die from background mortality do not release parasites because the parasites are either consumed or the latency period corresponds to the time necessary to develop viable progeny (Wang, 2006; White, 2011).

### Between-season dynamics

To study the impact of the feedback between host demography and parasite fitness on parasite evolution, we let the size of the emerging host cohort be a function of the number of uninfected hosts remaining at the end of the prior season. For the semelparous model:


$$\hat{s}\,\left(n+1\right)=\frac{\sigma \,\left(s_{n}\,\left(T\right)+\phi \,r_{n}\,\left(T\right)\right)}{1+\rho \,\left(s_{n}\,\left(T\right)+r_{n}\,\left(T\right)\right)}$$

For the iteroparous model:


$$\hat{s}\,\left(n+1\right)=\frac{\sigma \,\left(s_{n}\,\left(T\right)+\phi \,i_{n}\,\left(T\right)+\phi \,r_{n}\,\left(T\right)\right)}{1+\rho \,\left(s_{n}\,\left(T\right)+i_{n}\,\left(T\right)+r_{n}\,\left(T\right)\right)}$$

both of which correspond to Beverton–Holt growth, which is the discrete-time analogue of logistic growth in continuous time (Beverton & Holt, 1957). $s_{n}\,\left(T\right)$ is the density of susceptible hosts at t=T in season n, σ is host reproduction, ϕ is the reduction in fecundity experienced by hosts who are or have been infected, and ρ is the density-dependent parameter. We modelled host reproduction with negative density dependence as we assumed higher population density would reduce host fecundity due to, e.g., competition for resources.

We have shown previously that host carryover generates a feedback between parasite fitness and host demography that can select for quasiperiodic dynamics for some parameter ranges (MacDonald & Brisson, 2022). We explore the impact of parasite-induced increases in host mortality and decreases in host fecundity on the discrete-time dynamics in Appendix B.

### Parasite evolution

We use evolutionary invasion analysis (Geritz et al., 1998; Metz et al., 1992) to study how parasite latency periods adapt. We first extend system (1) to follow the invasion dynamics of a rare mutant parasite ($v_{n,m}$) in a population of resident parasites ($v_{n,r}$) in season n when parasite progeny transmission is synchronous following a latency period (τ):


$$\frac{d\,s_{n}}{d\,t}=\hat{s}\,\left(n\right)\,g\,\left(t,t_{l}\right)-\mu_{s}\,s_{n}\,\left(t\right)-\beta \,s_{n}\,\left(t\right)\,\left(v_{n,r}\,\left(t\right)+v_{n,m}\,\left(t\right)\right),$$ (3a)

$$\begin{array}{cc} \,\frac{d\,r_{n}}{d\,t} & =\beta (e^{-\mu_{s}\,\tau_{r}}s_{n}\left(t-\tau_{r}\right)v_{n,r}\left(t-\tau_{r}\right)+e^{-\mu_{s}\,\tau_{m}}s_{n}\left(t-\tau_{m}\right)\\ & \;v_{n,m}\left(t-\tau_{m}\right))-\mu_{r}r_{n}(t), \end{array}$$ (3b)

$$\begin{array}{cc} \frac{d\,v_{n,r}}{d\,t} & =\beta \,\alpha \,\left(\tau_{r},b\right)\,e^{-\mu \,\tau_{r}}\,s_{n}\,\left(t-\tau_{r}\right)\,v_{n,r}\,\left(t-\tau_{r}\right)-\delta \,v_{n,r}\,\left(t\right)\\ & \;-\beta \,s_{n}\,\left(t\right)\,v_{n,r}\,\left(t\right), \end{array}$$ (3c)

$$\begin{array}{cc} \frac{d\,v_{n,m}}{d\,t} & =\beta \,\alpha \,\left(\tau_{m},b\right)\,e^{-\mu \,\tau_{m}}\,s_{n}\,\left(t-\tau_{m}\right)\,v_{n,m}\,\left(t-\tau_{m}\right)\\ & \;-\delta \,v_{n,m}\,\left(t\right)-\beta \,s_{n}\,\left(t\right)\,v_{n,m}\,\left(t\right). \end{array}$$ (3d)

We also extend system (2) to follow parasite mutant invasion dynamics when parasite progeny transmission is distributed over time following a latency period (τ):


$$\frac{d\,s_{n}}{d\,t}=\hat{s}\,\left(n\right)\,g\,\left(t,t_{l}\right)-\mu_{s}\,s_{n}\,\left(t\right)-\beta \,s_{n}\,\left(t\right)\,\left(v_{n,r}\,\left(t\right)+v_{n,m}\,\left(t\right)\right),$$ (4a)

$$\;\,\frac{d\,i_{n,r}}{d\,t}=\beta \,e^{-\mu_{s}\,\tau_{r}}\,s_{n}\,\left(t-\tau_{r}\right)\,v_{n,r}\,\left(t-\tau_{r}\right)-i_{n,r}\,\left(t\right)\,\left(\mu_{i}+\gamma \right),$$ (4b)

$$\;\,\frac{d\,i_{n,m}}{d\,t}=\beta \,e^{-\mu_{s}\,\tau_{m}}\,s_{n}\,\left(t-\tau_{m}\right)\,v_{n,m}\,\left(t-\tau_{m}\right)-i_{n,m}\,\left(t\right)\,\left(\mu_{i}+\gamma \right),$$ (4c)

$$\;\,\frac{d\,r_{n}}{d\,t}=\gamma \,\left(i_{n,r}\,\left(t\right)+i_{n,m}\,\left(t\right)\right)-\mu_{r}\,r_{n}\,\left(t\right),$$ (4d)

$$\;\,\frac{d\,v_{n,r}}{d\,t}=\alpha \,\left(\tau_{r},b\right)\,i_{n,r}\,\left(t\right)-\delta \,v_{n,r}\,\left(t\right)-\beta \,s_{n}\,\left(t\right)\,v_{n,r}\,\left(t\right),$$ (4e)

$$\frac{d\,v_{n,m}}{d\,t}=\alpha \,\left(\tau_{m},b\right)\,i_{n,m}\,\left(t\right)-\delta \,v_{n,m}\,\left(t\right)-\beta \,s_{n}\,\left(t\right)\,v_{n,m}\,\left(t\right).$$ (4f)

where r and m subscripts refer to the resident and invading mutant parasites, respectively, and their corresponding traits.

In previous work on similar models that only considered parasites that complete one round of infection per season (monocyclic parasites), we were able to derive an analytical expression for mutant invasion fitness (MacDonald et al., 2022; MacDonald & Brisson, 2022). We are unable to solve the current models with parasites that complete multiple rounds of infection per season (polycyclic parasites) analytically due to the nonlinear $\alpha \,s_{n}\,\left(t\right)\,v_{n}\,\left(t\right)$ terms and instead determine parasite evolutionary end points numerically. Thus, for both models, we estimate the invasion fitness of rare mutants numerically. As in previous analyses (MacDonald & Brisson, 2022, 2023; MacDonald et al., 2022), the invasion fitness of a rare mutant parasite depends on the density of $v_{n,m}$ produced by the end of the season ($v_{n,m}\,\left(T\right)$) in the environment set by the resident parasite at equilibrium density ${\hat{v}}^{*}$. The mutant parasite invades in a given host phenological scenario if the density of $v_{n,m}$ produced by time T is greater than or equal to the initial $v_{n,m}\,\left(0\right)=1$ introduced at the start of the season ($v_{n,m}\,\left(T\right)\geq 1$).

The simulation analysis was done by first numerically simulating system (1) with a monomorphic parasite population with respect to the latency period (τ). A single mutant parasite is introduced at the beginning of the season after 100 seasons have passed. The mutant’s latency period strategy is drawn from a normal distribution whose mean is the value of τ from the resident strain. System (2) is then numerically simulated with the resident and mutant parasite. New mutants arise randomly after 1,000 seasons have passed since the last mutant was introduced, at which point system (2) expands to follow the dynamics of the new parasite strain. This new mutant has a latency period strategy drawn from a normal distribution whose mean is the value of τ from whichever parasite strain has the highest density. System (2) continues to expand for each new mutant randomly introduced after at least 1,000 seasons have passed. Any parasite whose density falls below 1 is considered extinct and is eliminated. The latency period evolves as the population of parasites with the adaptive strategy eventually invades and rises in density. Note that our simulations deviate from the adaptive dynamics literature in that new mutants can be introduced before earlier mutants have replaced the previous resident. Previous studies have shown that this approach is well suited to predicting evolutionary outcomes (Kisdi, 1999; MacDonald & Brisson, 2022; White & Bowers, 2005; White et al., 2006).

## Results

**Figure 2.**
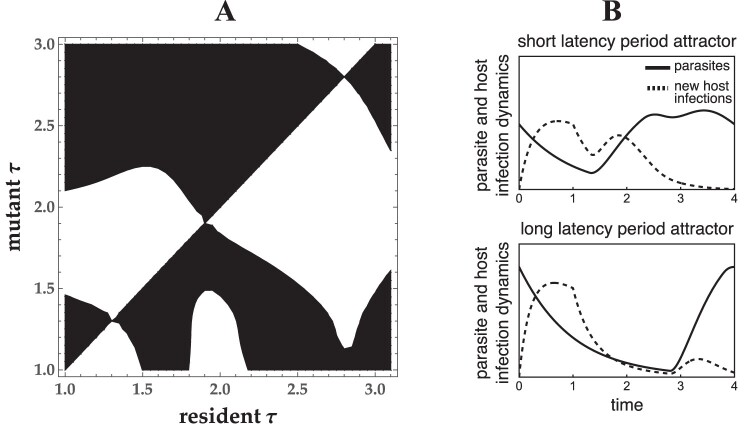
Seasonal host activity generates multiple parasite virulence attractors when parasite release is semelparous. (A) The pairwise invasibility plot (PIP) shows the outcome of invasion by mutant parasite strains into resident parasite populations that have latency period trait τ. Mutants possess an adaptive latency period trait and invade (black regions) or possess a maladaptive latency period trait and go extinct (white regions). The PIP shows two evolutionarily stable strategies (ESS) at τ=2.8 and τ=1.31 that are attractive and uninvasible for the parameter values used here. An evolutionary repellor lies between the two ESS at τ=1.9. (B) Top: Parasites with the shorter latency period phenotype (density shown by solid line, τ=1.31) complete two generations of infections during the season for the parameter values shown here and is thus polycyclic. Bottom: Parasites with the longer latency period phenotype (solid line, τ=2.8) release new parasites just prior to the end of the season and are thus monocyclic. Dashed line shows new host infections over time. $T=4,t_{l}=1,\alpha \,\left(\tau \right)=b\,{\left(\tau +0.5\right)}^{0.8}$. All other parameters are the same as in Table 1.

**Figure 3.**
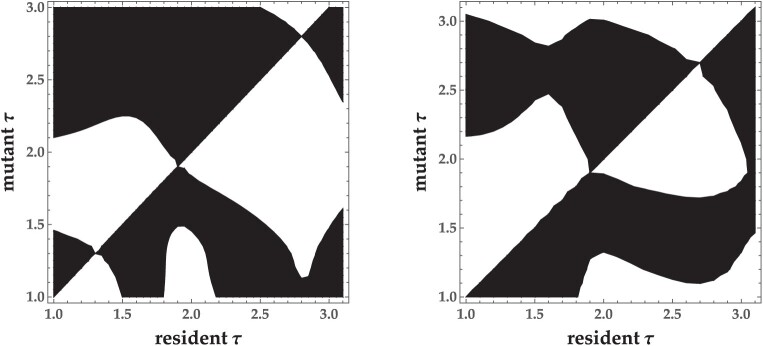
Host seasonality selects for parasite latency period bistability when the transmission of semelparous parasite progeny is (left) or is not (right) constrained by a trade-off between the number of progeny released and the length of the latency period. Left: $\alpha \,\left(\tau \right)=b\,{\left(\tau +0.5\right)}^{0.8},$ right: α=200, $T=4,t_{l}=1,$. All other parameters are the same as in Table 1.

**Figure 4.**
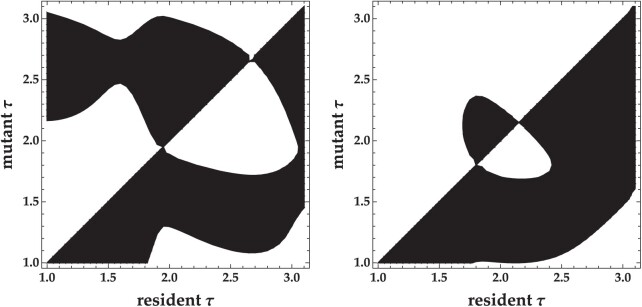
Host seasonality selects for parasite latency period bistability when the transmission of iteroparous parasite progeny is (left) or is not (right) constrained by a trade-off between the number of progeny released and the length of the latency period. Left: $\alpha \,\left(\tau \right)=b\,{\left(\tau +0.5\right)}^{0.8},$ right: α=400,$T=4,t_{l}=1$. All other parameters are the same as in Table 1.

Intermediate times between infection and parasite progeny release are adaptive for both obligate killer (MacDonald et al., 2022) and non-lethal, semelparous parasites (Figure 2). Similar to results for obligate killer parasites (MacDonald & Brisson, 2023), seasonal host activity also generates two evolutionarily stable strategies (ESSs) for non-obligate killer parasites: A shorter latency period strategy that allows multiple parasite generations within one season (polycylic transmission) and a longer latency period strategy that results in a single parasite generation each season (monocyclic transmission) (Figure 2). Furthermore, the model predicts that a semelparous life-history strategy, where hosts synchronously release parasite progeny, is not required for these results to hold (Figures 3 and 4). That is, intermediate latency periods are adaptive without a mechanistic trade-off, and evolutionary bistability is generated regardless of whether infected hosts synchronously release parasite progeny (semelparous) or release progeny over a longer period of time(iteroparous).

### Lethal vs. non-lethal parasites

**Figure 5.**
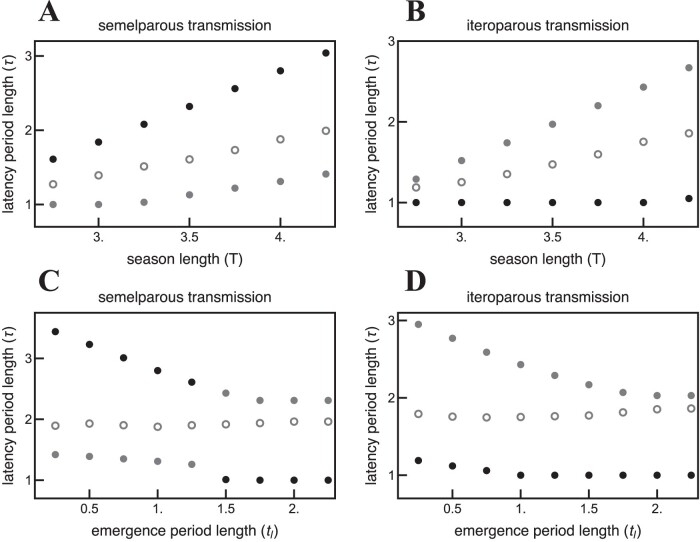
Host seasonality selects for latency period bistability for parasites when the transmission of parasite progeny is semelparous (A and C) or iteroparous (B and D). Furthermore, semelparous parasite populations at the longer latency period ESS generally outcompete parasite populations at the shorter latency period ESS. However, this trend is reversed for iteroparous parasites. Black points indicate the evolutionary attractor that outcompetes the other ESS (i.e., global attractor); gray points indicate local attractors; hollow points indicate repellors. $T=4,t_{l}=1,\alpha \,\left(\tau \right)=b\,{\left(\tau +0.5\right)}^{0.8}$. All other parameters are the same as in Table 1.

Seasonal host activity generates both a shorter latency period ESS and a longer latency period ESS for semelparous parasites, regardless of whether they are obligate killers (MacDonald et al., 2022, Figure 2). Shorter and longer latency period ESSs are separated by an evolutionary repellor such that the two strategies cannot coexist in the same environment. The ESS that a semelparous parasite population evolves towards is determined by the latency period of the initial resident population. Host phenological patterns determine both ESSs: shorter activity periods and longer host emergence periods select for shorter latency times (Figure 5A and C), as seen previously for obligate killer parasites (MacDonald et al., 2022). The longer latency period ESS reaches an intermediate trait value in the absence of a mechanistic trade-off between transmission and the time between infection and new parasite release, analogous to previous results for obligate-killer parasites (Figure 2 in MacDonald et al. 2022). In the absence of a trade-off, however, the shorter latency period ESS always corresponds to the minimum possible latency period (Figures 3 and 4).

### Semelparous vs. iteroparous for non-lethal parasites

**Figure 6.**
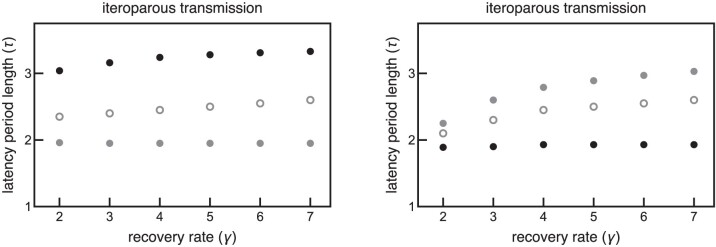
The left plot shows that short latency period strategies dominate for iteroparous parasites when the host emergence period in short ($t_{l}=0.5$), while the right plot shows that long latency period strategies dominate when the host emergence period is long ($t_{l}=2$). These results demonstrate that the results of the iteroparous model approach those of the semelparous model as the transmission and recovery rates increase. $\beta =10^{-5}$. All other parameters are the same as in Table 1.

Host phenology selects for qualitatively similar ESS virulence strategies for non-lethal semelparous and iteroparous parasites. That is, host phenology selects for both a shorter latency period ESS and a longer latency period ESS separated by an evolutionary repellor, regardless of whether parasites are semelparous or iteroparous (Figure 5). However, semelparous and iteroparous parasites have quantitatively different ESS latency periods. Semelparity selects for longer latency periods such that the release of all parasites occurs just before the end of the season (Figure 5A and C). Conversely, iteroparity selects for shorter latency periods in order to ensure infected hosts have time to release parasites before the end of the season (Figure 5B and D). Furthermore, parasites with the longer latency period ESS generally outcompete parasites with the shorter latency period ESS for semelparous parasites (Figure 5A and C), while this trend is reversed for iteroparous parasites (Figure 5B and D). The results of the iteroparous model more closely match the results of the semelparous model as the transmission rate and recovery rate increase. Figure 6 shows that the long latency period strategy dominates in the iteroparous model when the emergence period is short and the short latency period strategy dominates when the emergence period is long. These results are qualitatively similar to what is presented in Figure 5C for semelparous parasites.

**Figure 7.**
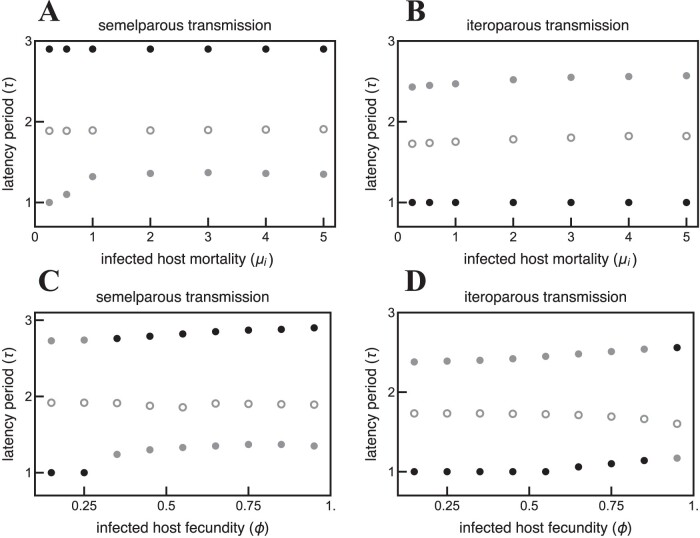
Parasite-induced increases in host mortality ($\mu_{i}$) or decreases in fecundity (ϕ) have minimal impact on parasite latency period evolution (τ). (A) Low infected host mortality rates select for slightly shorter latency periods for the shorter latency period ESS but have no impact on the longer latency period ESS for semelparous parasites. (B) Conversely, low infected host mortality rates select for slightly shorter latency periods for the longer τ ESS but have no impact on the shorter τ ESS for iteroparous parasites. (C) Low parasite-induced infected host fecundity selects for shorter semelparous latency periods for the shorter τ ESS but have minimal impact on the longer τ ESS. (D) In contrast, when iteroparous parasites minimally reduce infected host fecundity, slightly longer latency periods are adaptive for both the shorter τ ESS and the longer τ ESS. Black points indicate the global attractor; gray points indicate local attractors; hollow points indicate repellors. $T=4,t_{l}=1,\alpha \,\left(\tau \right)=b\,{\left(\tau +0.5\right)}^{0.8}$. All other parameters are the same as in Table 1.

The rate of parasite-induced host mortality has only a small impact on the optimal latency periods of semelparous and iteroparous parasites (Figure 7A and B). Shorter latency periods are adaptive for non-obligate killer parasites when parasite-induced host mortality is low; however, the impact varies depending on whether parasites are semelparous or iteroparous. Low parasite-induced host mortality rates select for slightly shorter latency periods for the *shorter latency period* ESS but have no impact on the *longer latency period* ESS for semelparous parasites (Figure 7A). Conversely, low infected host mortality rates select for slightly shorter latency periods for the *monocyclic* ESS but have no impact on the *polycyclic* ESS for iteroparous parasites(Figure 7B).

The impact of parasite infection on host fecundity has a small impact on optimal latency periods for semelparous and iteroparous parasites (Figure 7C and D). Shorter latency periods are adaptive for parasites that strongly decrease host fecundity, as opposed to killing the host; however, the effect is small. Decreased host fecundity decreases equilibrium host densities, which shifts the timing of infections later in the season because transmission is density dependent (Equations 1a, 2a, 3a, and 4a). Infections that occur later in the season select for shorter latency periods to ensure that parasite progeny are released before the end of the season. The impact of infection-induced reductions in host fecundity has qualitatively different impacts on semelparous and iteroparous parasites. That is, the longer latency period ESS is the semelparous parasite global attractor for a greater range of infected host fecundities compared to iteroparous parasites.

## Discussion

The assumption that parasites must be obligate-host killers is not necessary for seasonal host activity to select for intermediate latency periods in the absence of a trade-off between latency period length and number of parasite progeny (Figure 2). Furthermore, seasonal host activity can select for two evolutionary stable strategies when parasites are obligate-killers (MacDonald & Brisson, 2023) and are not obligate-killers: a shorter latency period ESS and a longer latency period ESS (Figure 5). Finally, seasonal host activity patterns select for intermediate latency periods in both semelparous parasites that release all progeny simultaneously and iteroparous parasites that release progeny over a longer period of time. These results suggest that seasonal host activity can be an important driver of parasite life-history strategies in a wider range of parasites than previously recognized (MacDonald & Brisson, 2023; MacDonald et al., 2022). While the model would need to be altered to fit any specific system, this general model can serve as the foundation to study obligate-killer parasites (Baltensweiler et al., 1977; Donovan, 1991; Grant & Shepard, 1984; Takasuka & Tanaka, 2013), non-lethal parasites (Crowell, 1934; Gaulin et al., 2007; Li et al., 2007; Zhang & Fernando, 2017; Zehr, 1982), semelparous parasites (Baltensweiler et al., 1977; Grant & Shepard, 1984), or iteroparous parasites (Gaulin et al., 2007; Kakaire et al., 2012; Zehr, 1982).

The results presented here are qualitatively similar to theory developed for latency period evolution of obligate-killer parasites in seasonal environments (MacDonald et al., 2022). Seasonal host activity sets up an alternative trade-off between releasing new parasites too early or too late regardless of whether the parasite must kill its host to release progeny. For both obligate-killer and non-obligate killer parasites, longer latency periods are maladaptive for parasites as they do not release progeny before the end of the season when all adult hosts die regardless of their infection status. Shorter latency periods are also maladaptive in both cases as progeny released early are more likely to die due to environmental exposure. Thus, the conflicting costs of not releasing progeny before the end of the season (which results in zero new progeny) and releasing progeny too early in the season (which results in many progeny dying from environmental exposure) selects for intermediate latency periods. Taken together, these results suggest that parasites need not kill their host for seasonality to make intermediate latency periods adaptive.

Parasite transmission strategies impact the evolution of latency period quantitatively, but not qualitatively. Iteroparous parasites that release progeny over time have shorter optimal latency periods than semelparous parasites that release progeny all at once (Figure 5). Iteroparous parasites require shorter latency periods to increase the number of progeny released before the end of the season. Conversely, semelparous parasites require longer latency periods to decrease the number of progeny that decay in the environment before the end of the season. The longer latency period optimum tends to outcompete the shorter latency period optimum for semelparous parasites, while the converse is true for iteroparous parasites. The model thus predicts that semelparous parasites found in nature are likely to have longer latency periods, while iteroparous parasites are likely to have shorter latency periods.

Several features of the current model can be altered to investigate more complex impacts of host phenology on parasite latency period evolution. For example, the model presented here could be extended to study the impact of different host reproductive strategies on parasite latency period evolution by allowing hosts to reproduce more than once per season. Hosts that reproduce throughout the season would likely favour shorter latency period strategies that rely on hosts being available mid-season for later parasite generations (van den Berg et al., 2011). The model could also be used to study the impact of different types of trade-offs between parasite latency period and other traits, such as the mortality rate of hosts that recovered from infection. However, increased host mortality following parasite infection is not predicted to strongly impact optimal latency periods in the current framework (Figure 7).

**Figure 8.**
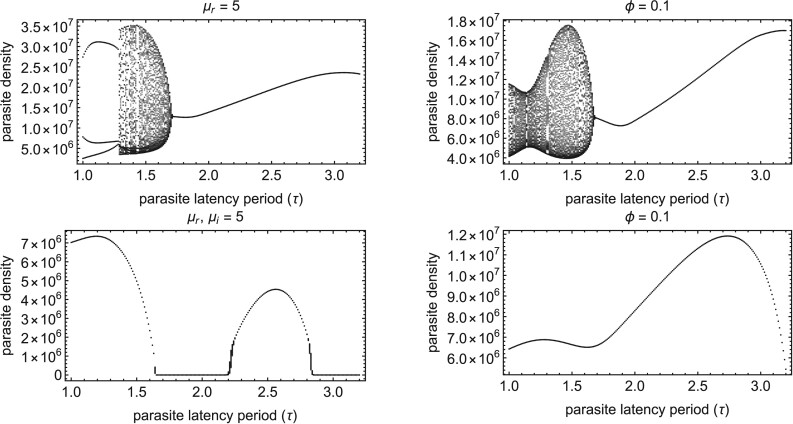
Semelparous parasites are more likely to drive host–parasite demographic cycling than iteroparous parasites. The top panel shows the semelparous parasite discrete-time dynamics, and the bottom panel shows iteroparous parasite discrete-time dynamics for seasons 400–500 (i.e., when the system is at its ecological attractor). The panels on the left demonstrate that high parasite-induced host mortality can drive cycling when parasites are semelparous, but not iteroparous. The panels on the right demonstrate that large parasite-induced decreases in host fecundity can drive cycling when parasites are semelparous, but not iteroparous. $\mu_{r}=\mu_{i}=5,\phi =0.1$. All other parameters are the same as in Table 1.

Host phenology impacts the timing and prevalence of transmission opportunities for parasites (Altizer et al., 2006; Biere & Honders, 1996; Gethings et al., 2015; Hamer et al., 2012; Ogden et al., 2018; Martinez, 2018; MacDonald et al., 2020; McDevitt-Galles et al., 2020), which selects parasite life-history strategies (Donnelly et al., 2013; Hamelin et al., 2011; King et al., 2009; MacDonald et al., 2022; MacDonald & Brisson, 2023; van den Berg et al., 2011). Past work has shown that host phenology can select for intermediate latency periods and select for multiple evolutionarily stable parasite strategies, but only in obligate-killer parasites (MacDonald et al., 2022; MacDonald & Brisson, 2023). The present study extends this area of research by predicting that host phenology can also select for intermediate latency periods and multiple evolutionarily stable strategies in non-lethal parasites. Thus, seasonal host patterns could act as a selective force in a wide range of disease systems given that non-lethal parasites are extremely common in nature.

## Data availability

Code is available on the Github repository: https://github.com/hanneloremac/intermediate-latency-strategies.

## Author contributions

H.M. conceived of the presented idea and developed the theoretical framework, conducted the mathematical analysis, and performed numerical simulations; both authors wrote the manuscript and gave final approval for publication and agree to be held accountable for the work performed therein.

## Funding

This work was supported by the National Institutes of Health (T32AI141393 [H.M.] R01AI142572 [D.B.], R01AI097137 [D.B.]; the National Science Foundation (DEB-1354184 [D.B.]; and the Burroughs Wellcome Fund (1012376 [D.B.]).

## Conflicts of interest

The authors declare no conflicts of interest.

## Appendix A

In Appendix A, we describe the numerical methods used to generate PIPs and to find evolutionary attractors and repellors.

We follow the same approach as previous work (MacDonald et al., 2022; MacDonald & Brisson, 2022) and define mutant invasion fitness as the density of mutant parasites produced by the end of the season ($v_{n,m}\,\left(T\right)$) in the environment set by the resident parasite at equilibrium density ${\hat{v}}^{*}$. The mutant parasite invades if the density of $v_{n,m}$ produced by time T is greater than or equal to the initial $v_{n,m}\,\left(0\right)=1$ introduced at the start of the season ($v_{n,m}\,\left(T\right)\geq 1$). Note that the mutant will not coexist with the resident strain when $v_{n,m}\,\left(T\right)>1$ given that there is an evolutionary repellor in between the two ESSs instead of an evolutionary branching point (Geritz et al., 1998; Metz et al., 1992; Waxman & Gavrilets, 2005).

It is not possible to derive an algebraic expression for mutant invasion fitness in this study as it was in previous studies. To generate PIP plots for pairs of resident’s with virulence ($\tau_{r}$) and mutant’s with virulence ($\tau_{m}$), we instead numerically find the density of $v_{n,m}\,\left(T\right)$ after one season in an environment set by the resident parasite, $v_{n}$. As in the previous analytical approach (MacDonald et al., 2022; MacDonald & Brisson, 2022, 2023), $v_{n,m}\,\left(T\right)=1$ corresponds to a neutral mutant, $v_{n,m}\,\left(T\right)>1$ corresponds to a mutant-resident pair in which the mutant parasite can invade and replace the resident, $v_{n,m}\,\left(T\right)<1$ corresponds to a mutant-resident pair that drives the mutant parasite extinct.

We use a similar approach to locate virulence trait values (τ) that correspond to evolutionary attractors and repellors. We again numerically find $v_{n,m}\,\left(T\right)$ after one season in an environment set by the resident $v_{n}$. Values of τ corresponding to attractors and repellors prevent small effect mutants with higher and lower virulence from invading. That is, when resident virulence is $\tau_{r}$, mutants with $\tau_{m}=\tau_{r}+0.01$ and $\tau_{m}=\tau_{r}-0.01$ cannot invade. We determine which points are attractors and repellors if there is more than one virulence trait value that prevents mutant invasion. Repellors are always found in between two attractors. To determine their location, we find the value of τ in between the two attractors that correspond to a minimum for $v_{n,m}\,\left(T\right)$.

To determine which attractor is the global attractor, we find the attractor that competitively excludes all others. Mutant parasites with the value of τ corresponding to the global attractor can invade a population of resident parasites with the value of τ corresponding to non-global, local attractors. Resident parasites with the value of τ corresponding to the global attractor also prevent invasion of a mutant parasite with the value of τ corresponding to non-global, local attractors.

## Appendix B

Parasites can overexploit host populations resulting in demographic cycling for some parameters.

Non-lethal parasites are less likely to drive cyclical host–parasite dynamics than obligate-killer parasites. That is, cycling occurred in a narrower parameter range compared to previous work on a similar model (MacDonald & Brisson, 2022). In the present study, parasite infections either need to result in large decreases in host fecundity or large increases in host mortality following infection to drive cycling host–parasite dynamics (Figure 8). Semelparous parasites with adaptive latency periods impact host populations more strongly than iteroparous parasites and are thus more likely to drive cycling.

Non-lethal parasites are less likely to drive cycling than lethal parasites, in line with previous results (e.g., Hilker et al. 2020). That is, seasonal disease systems with non-lethal parasites are less likely to display cyclic population dynamics than disease systems with obligate-killer parasites. These results suggest that non-lethal parasites will adapt more quickly on average in nature as they are less prone to drive cycling population dynamics, which has been previously shown to decrease the rate of adaptation through an eco-evolutionary feedback (MacDonald & Brisson, 2022).

## Appendix C

The results of the iteroparous model become more similar to the semelparous model when the transmission rate and recovery rate increase.
